# Supplementary material for: Computer-Aided Prediction, Synthesis, and Characterization of Magnetic Molecularly Imprinted Polymers for the Extraction and Determination of Tolfenpyrad in Lettuce
Source: Foods. 2023 Mar 1;12(5):1045. doi: 10.3390/foods12051045 (PMC10001402; doi:10.3390/foods12051045)
Supplement: Supplementary file 1 [file foods-12-01045-s001.zip › foods-2236332-supplementary.pdf]

Supplementary Information

Table S1. Infrared wavenumbers of tolfenpyrad at the B3LYP, PBEPBE and ωB97XD levels with 6-31G (d, p) and 6-31G+ (d, p) basic sets and available experimental (EXP) data.

**Species**	**6-31G (d, p)**			**6-31G+ (d, p)**			**EXP**
	**B3LYP**	**PBEPBE**	**ωB97XD**	**B3LYP**	**PBEPBE**	**ωB97XD**	
30	454.08	441.23	461.33	451.22	437.58	459.84	432.03
32	496.08	486.82	511.47	506.03	489.08	513.65	505.01
35	560.86	542.82	578.26	560.66	540.77	575.89	579.54
36	590.05	568.63	608.72	588.42	566.47	609.16	590.08
38	622.26	602.63	628.84	618.90	600.68	627.75	616.67
44	709.55	690.26	721.27	701.01	684.08	715.42	673.46
47	759.10	737.94	775.07	770.24	737.94	780.38	756.30
50	807.24	784.91	821.02	867.10	791.29	818.06	790.79
51	832.15	799.36	846.74	828.38	797.69	844.23	813.82
54	848.89	815.76	876.44	846.86	809.49	873.18	845.43
55	870.99	840.64	889.44	867.10	838.02	885.56	858.07
56	894.69	872.66	908.10	891.57	869.66	905.15	874.00
60	966.31	940.33	998.85	974.10	934.78	999.66	933.79
65	1034.97	1002.93	1046.48	1032.98	1001.3	1045.05	992.53
67	1052.29	1024.05	1073.64	1050.04	1020.93	1075.21	1011.96
75	1197.02	1159.61	1217.35	1194.16	1156.50	1213.46	1102.84
81	1275.21	1238.58	1288.29	1266.25	1225.13	1285.51	1164.70
82	1289.10	1248.63	1309.40	1278.37	1243.59	1301.26	1234.25
84	1308.19	1273.80	1332.41	1301.86	1268.05	1325.89	1286.04
89	1369.39	1350.42	1387.71	1364.58	1345.77	1382.41	1355.51
104	1546.36	1496.67	1572.67	1538.91	1489.76	1565.55	1497.30
106	1566.99	1513.45	1597.04	1563.49	1510.17	1592.91	1506.66
107	1596.94	1546.68	1637.51	1590.75	1540.71	1632.60	1551.56
110	1661.02	1615.93	1699.80	1651.47	1607.29	1691.64	1603.06
112	1737.93	1687.11	1791.71	1711.98	1663.64	1765.83	1647.95
114	3036.61	2976.91	3066.03	3033.23	2973.42	3063.10	2875.34
116	3059.64	2998.02	3099.81	3053.37	2992.15	3095.40	2933.32
117	3067.84	3000.00	3100.27	3062.99	2992.94	3096.47	2972.92
118	3096.79	3043.22	3138.20	3091.72	3037.29	3133.12	3035.73
121	3132.65	3081.53	3166.99	3129.14	3071.15	3160.23	3293.00

Table S2. Isotherm parameters for the binding of tolfenpyrad on MNIPs and MMIPs.

Sample	Q_e (mg/g)	Langmuir			Freundlich			
		Q_{max} (mg/g)	K_L (L/mg)	R^2	Q_{max} (mg/g)	K_F (L/mg)	R^2	n
MNIPs	1.25	1.81	0.018	0.924	1.15	0.009	0.992	1.81
MMIPs	7.20	17.39	0.008	0.944	8.65	0.227	0.997	1.26

Table S3. Kinetics parameters for the binding of tolfenpyrad on MNIPs and MMIPs.

Sample	Q_e (mg/g)	Pseudo-first-order			Pseudo-second-order		
		Q_{1c} (mg/g)	K_1 (min^{-1})	R^2	Q_{2c} (mg/g)	K_2 (min^{-1})	R^2
MNIPs	1.25	1.47	0.051	0.968	1.30	0.055	0.992
MMIPs	7.20	7.92	0.050	0.964	7.86	0.101	0.995

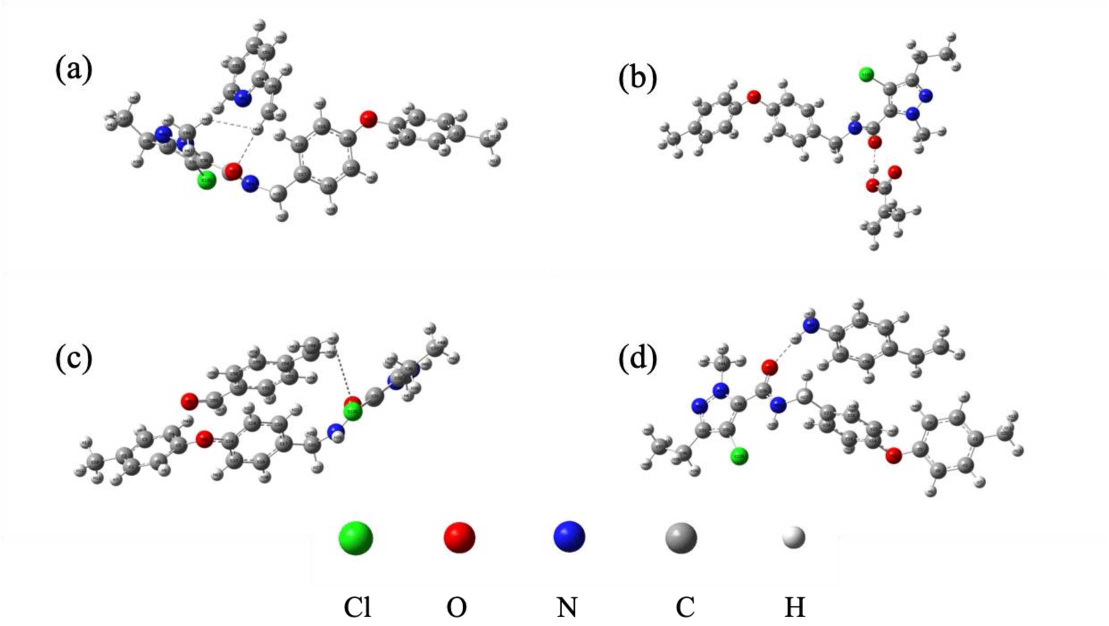

Figure S1. Interactions of the tolfenpyrad-monomer (**a**) tolfenpyrad-2-VA, (**b**) tolfenpyrad-MAA, (**c**) tolfenpyrad-4-VB and (**d**) tolfenpyrad-4-VA.

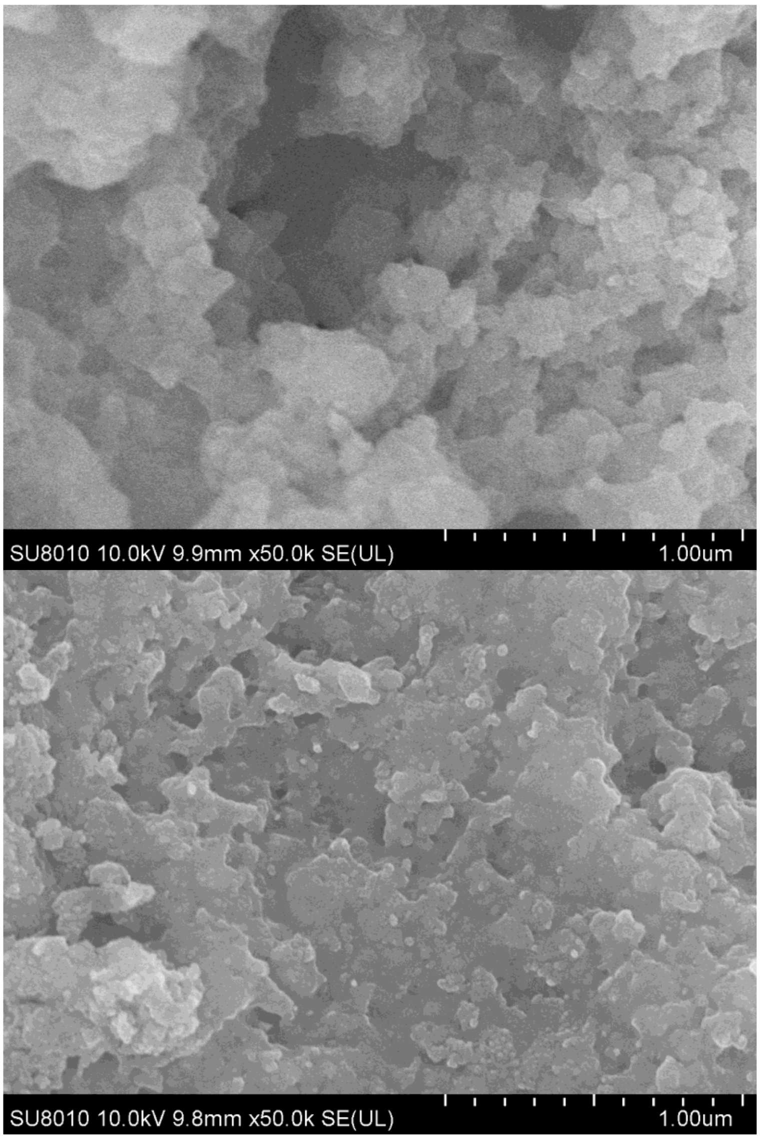

Figure S2. SEM micrographs of MMIP (**A**) and MNIP (**B**) (Magnification scale at 50,000×).
